# The effects of the COVID-19 pandemic on psychological stress in breast cancer patients

**DOI:** 10.1186/s12885-021-09012-y

**Published:** 2021-12-31

**Authors:** Catharina Bartmann, Leah-Maria Fischer, Theresa Hübner, Max Müller-Reiter, Achim Wöckel, Rhiannon V. McNeill, Tanja Schlaiss, Sarah Kittel-Schneider, Ulrike Kämmerer, Joachim Diessner

**Affiliations:** 1 Department of Obstetrics and Gynaecology, University Hospital of Würzburg, Josef-Schneider-Str. 4, Würzburg, 97080 Germany; 2 Department of Psychiatry, Psychosomatic Medicine and Psychotherapy, University Hospital of Würzburg, Margarete-Höppel-Platz 1, Würzburg, 97080 Germany

**Keywords:** COVID-19, Breast Cancer, Psychological distress

## Abstract

**Background:**

The majority of breast cancer patients are severely psychologically affected by breast cancer diagnosis and subsequent therapeutic procedures. The COVID-19 pandemic and associated restrictions on public life have additionally caused significant psychological distress for much of the population. It is therefore plausible that breast cancer patients might be particularly susceptible to the additional psychological stress caused by the pandemic, increasing suffering. In this study we therefore aimed to assess the level of psychological distress currently experienced by a defined group of breast cancer patients in our breast cancer centre, compared to distress levels pre-COVID-19 pandemic.

**Methods:**

Female breast cancer patients of all ages receiving either adjuvant, neoadjuvant, or palliative therapies were recruited for the study. All patients were screened for current or previous COVID-19 infection. The participants completed a self-designed COVID-19 pandemic questionnaire, the Stress and Coping Inventory (SCI), the National Comprehensive Cancer Network® (NCCN®) Distress Thermometer (DT), the European Organization for Research and Treatment of Cancer (EORTC) QLQ C30, and the BR23.

**Results:**

Eighty-two breast cancer patients were included. Therapy status and social demographic factors did not have a significant effect on the distress caused by the COVID-19 pandemic. The results of the DT pre and during COVID-19 pandemic did not differ significantly. Using the self-designed COVID-19 pandemic questionnaire, we detected three distinct subgroups demonstrating different levels of concerns in relation to SARS-CoV-2. The subgroup with the highest levels of concern reported significantly decreased life quality, related parameters and symptoms.

**Conclusions:**

This monocentric study demonstrated that the COVID-19 pandemic significantly affected psychological health in a subpopulation of breast cancer patients. The application of a self-created “COVID-19 pandemic questionnaire” could potentially be used to help identify breast cancer patients who are susceptible to increased psychological distress due to the COVID-19 pandemic, and therefore may need additional intensive psychological support.

**Trial registration:**

DRKS-ID: DRKS00022507.

**Supplementary Information:**

The online version contains supplementary material available at 10.1186/s12885-021-09012-y.

## Background

The pandemic spread of the novel Coronavirus SARS-CoV-2, resulting in Coronavirus disease 2019 (COVID-19), poses extreme challenges to public health systems in many countries. Hospitals in some regions of the world are inundated with patients with respiratory symptoms, which has induced a fear of overcrowding and lack of access to healthcare in the population. A current major focus of medical institutions is therefore on the management of those patients presenting with acute disease and infection symptoms [1]. The outbreak of COVID-19 has also caused mental health stress for a large proportion of the population [2]. People suffering from cancer might be particularly affected by the COVID-19 pandemic, as they already typically experience a loss of certainty, stress, anxiety and depression as a result of cancer diagnosis and treatment [3, 4]. Moreover, cancer patients are significantly more vulnerable to infection and poor infection outcomes, due to immunosuppression [5]. The COVID-19 pandemic has additionally negatively affected the performance of the medical system, particularly for tumour patients, as medical logistics are currently focused on treating COVID-19 patients [6, 7]. Cancer patients are also usually able to develop coping strategies because of direct contact with other cancer patients undergoing treatment, and regular contact with clinical psychiatrists, psychologists and self-help groups. However, contact with these sources of help has been inhibited due to the COVID-19 pandemic. For cancer patients the COVID-19 pandemic therefore presents an extreme additional psychological stress factor that should not be ignored by health services [6, 7].

Many current psychological studies address the mental health of the general population or medical personnel directly involved in the treatment of COVID-19 patients [4], whereas data on the potential psychological impact of the COVID-19 pandemic on cancer patients is scarce. This is an area of concern, as pre-pandemic up to 10% of cancer patients were already reported to develop clinically relevant depression symptoms, whether in curative or palliative treatment [8]. Moreover, depression is associated with poor treatment adherence and reduced cancer survival [7–9]. Patients with gynaecological tumours and lung cancer are particularly susceptible to depression, possibly due to pain levels, prognosis, and body image disruption associated with different tumour types [8, 10, 11].

Breast cancer is the most common malignancy among women, with an incidence of approximately 70.000 cases per year in German [12]. The number of clinical research studies with a focus on the psychological influence of COVID-19 and the COVID-19 pandemic on breast cancer patients is limited [13–15]. To address this, we conducted a survey on breast cancer patients undergoing different therapy treatments during the first wave of the COVID-19 pandemic, based in our cancer centre in Würzburg (Germany). To evaluate the psychological impact of the pandemic on breast cancer patients we used several self-report questionnaires; our self-designed COVID-19 pandemic questionnaire, the Stress and Coping Inventory (SCI), the NCCN Distress Thermometer (DT) of the National Comprehensive Cancer Network® (NCCN®), the European Organisation for Research and Treatment of Cancer (EORTC) QLQ-C30 (version 3.0) and the EORTC QLQ-BR23 [16–21]. During treatment, distress levels are routinely monitored for all cancer patients, in order to set up appropriate interventions if necessary. Thus, we were able to retrospectively analyze patient distress levels before the spread of SARS-CoV-2 and approximately 2 months after the government enforced isolation and lockdown sanctions in the same group of patients. This unique situation allowed us to assess the additional psychological stress caused by the COVID-19 pandemic in breast cancer patients.

## Methods

### Materials

#### COVID-19 pandemic questionnaire

Our self-designed COVID-19 pandemic questionnaire consists of two parts. In the first part, there are questions regarding infection during the last 4 months. The open-ended introductory question screens for an infection and infection type. The following symptoms are queried using a yes/no-answer; fever (temperature higher than 38.5 °C), cough, shortness of breath, muscle and joint pain, sore throat, headache, nausea/vomiting, nasal congestion, diarrhoea, taste and/or odour disorders and pneumonia. This is followed by questions about possible contact with a SARS-CoV-2 positive person, or if there has been a historical positive throat swab for SARS-CoV-2 and / or a COVID-19 disease.

In the second part, questions 1–8 (Table 1) regarding concerns surrounding the COVID-19 pandemic were answered using the following Likert scale: 1 = No, never; 2 = I have thought about it, but was not worried; 3 = I am a little concerned; 4 = I am often concerned; 5 = I am concerned about it all the time. In questions 9–11 (Table 1) the Likert scale was modified: 1 = not at all; 2 = a little bit, 3 = moderately; 4 = quite a lot; 5 = a lot.

**Table 1:** Questions, scales and results of the COVID-19 pandemic questionnaire answered by 82 breast cancer patients. Median and interquartile range of the following ordinal scale (1–5) are presented

		Median	Interquartile range
Concern scale	1. Are/were you concerned about infecting yourself with SARS-CoV-2?	2.00	2.00–3.00
	2. Are/were you concerned about becoming infected during doctor and hospital visits?	2.00	2.00–3.00
	3. Are/were you concerned about your family becoming infected with SARS—CoV-2?	2.00	1.00–3.00
	4. Are/were you worried about dying from COVID-19?	3.00	2.00–3.00
Concern over time scale	5. Are/were you concerned about becoming infected when the first European patient was reported?	2.00	1.00–2.00
	6. Were you concerned about becoming infected when the first European patient died?	2.00	1.00–2.00
	7. Were you concerned about becoming infected when the number of infected people increased?	3.00	2.00–3.00
	8. Were you concerned about becoming infected at the lockdown?	2.00	2.00–3.00
Impairment scale	9. How much is your quality of life affected by the COVID-19 pandemic?	3.00	2.00–4.00
	10. How much is the breast cancer therapy affected by the COVID-19 pandemic?	3.00	2.00–4.00
	11. How much is your mental health affected by the COVID-19 pandemic?	2.00	2.00–4.00

For questions 1–8: 1 = no, never; 2 = I have thought about it, but was not worried; 3 = I am a little concerned; 4 = I am often concerned; 5 = I am concerned about it all the time

For questions 9–11: 1 = not at all; 2 = a little bit, 3 = moderately; 4 = quite a lot; 5 = a lot

#### Stress and coping inventory (SCI)

The stress and coping inventory is a German-language questionnaire with 54 items to record stress levels, stress symptoms and coping strategies [18]. The initial normal sample from 2012 included 5220 participants with a Cronbach’s Alpha 0.69 to 0.88. The first 21 items of the SCI detect the total stress level with a seven-point Likert scale from “not burdened” to “very heavily burdened”. The total stress level is divided into three subscales consisting of seven items each; “stress caused by insecurity”, “stress caused by being overwhelmed”, and “stress caused by loss”. Physical stress symptoms are queried in the following 13 items. A four point Likert scale is again used; “does not apply at all”, “applies a bit”, “moderately applies” and “applies completely”. The same scale is used to record coping, each with four items. Coping is divided into “positive coping”, “active coping”, “coping by support”, “coping by believing in God or powers that be” and “coping by drinking alcohol and/or smoking”. For the evaluation of stress, stress symptom and coping scales, the individual point values were added as provided in the evaluation manual [19].

#### Distress thermometer (DT)

The NCCN Distress thermometer (DT) of the National Comprehensive Cancer Network® (NCCN®) was first described by Holland in 1997, and is a clinical standard tool to measure the distress of cancer patients [16]. Based on a temperature scale from “0 = not burdened at all” to “10 = extremely burdened”, current distress levels can be measured. The German version was used in our study [20].

#### EORTC QLQ-C30 and QLQ-BR23

The European Organization for Research and Treatment of Cancer (EORTC) QLQ-C30 version 3.0 questionnaire, and the QLQ-BR23 breast cancer-specific module, were also used to assess quality of life. Both questionnaires consist of single items and multi-item scales. The EORTC QLQ C30 contains 30 items representing “global health status/quality of life (QL2)”; the functional scales “physical functioning (PF2)”, “role functioning (RF)”, “emotional functioning (EF)”, “cognitive functioning (CF)”, and “social functioning (SF)”; the symptom scales “fatigue (FA)”, “nausea and vomiting (NV)”, and “pain (PA)”; and the symptom items “dyspnoea (DY)”, “insomnia (SL)”, “appetite loss (AP)”, “constipation (CO)”, “diarrhoea (DI)”, and “financial difficulties (FI)”. The QLQ-BR23 consists of 23 breast cancer-specific questions, which make up the following categories; the functional scales “body image (BRBI)”, “sexual functioning (BRSEF)”, sexual enjoyment (BRSEE)”, and “future perspective (BRFU)”; and the symptom scales/items “systemic therapy side effects (BRST)”, “breast symptoms (BRBS)”, “arm symptoms (BRAS)”, and “upset by hair loss (BRHL)” [17]. All single items and multi-items scales were calculated in percent with a range from 0 to 100, according to the scoring manual [21, 22].

### Ethics approval

All investigations were approved by the Ethics committee of the University of Würzburg (No. 70/20 Amendment). All participants agreed to participate in the study with written informed consent after receiving verbal and written information.

### Study site

The oncological section at the Department of Obstetrics and Gynaecology, University Hospital Würzburg, is located in Würzburg, a city with 130.000 inhabitants with a large rural catchment area, and belongs to the Comprehensive Cancer Center (CCC) Mainfranken [23], which is just one of 14 interdisciplinary oncology centers of excellence in Germany.

The CCC Mainfranken is located in Franconia (23,000 km2) and treats about 4500 cancer patients per year. Regarding breast cancer, there are more than 300 patients with primary diagnosis and about 300 patients with metastatic disease annually.

### Participants

Inclusion criteria were breast cancer diagnosis, age of 18–80 years, no known current infection with SARS-CoV-2, sufficient knowledge of German and the ability to give consent. Breast cancer patients who met the inclusion criteria and were receiving either sequencing endocrine therapy, targeted therapy, and/or chemotherapy at the time of the study were asked to participate in the study. Cancer therapy included neoadjuvant, adjuvant and palliative treatments. Patients were treated on an outpatient or inpatient basis in the gynaecological clinic of the Würzburg University Hospital, from April to June 2020. Patients were recruited via regular visits to the hospital as part of their standard treatment, and were not compensated for participation in the study. The first diagnosis of breast cancer ranged from recent (3 weeks before the time of recruitment) to several years previously (9 years before the study start). Some patients were therefore undergoing initial therapy, whereas others were receiving different palliative treatments (not end of life) with the aim of extending life or limiting symptoms. After receiving detailed information about the study, patients willing to participate gave written informed consent and were allocated a study number, which was then documented on their corresponding clinical records.

### Procedure

Qualifying breast cancer patients receiving sequencing endocrine therapy, targeted therapy and/or chemotherapy in a neoadjuvant, adjuvant or palliative setting were asked to take part in this study. Patients received written and verbal study information, and then gave written informed consent. Each patient was allocated a study number, underwent a throat swab for SARS-CoV-2, Anti-SARS-CoV-2 antibody titre was also measured, as part of routine blood tests. Self-report questionnaires were completed by the patients during their cancer treatment in the treatment and/or hospital rooms. An overview of the patient recruitment and data collection is shown in Supplemental Fig. 1.

### Laboratory analysis

Patient throat swabs were taken as part of the routine clinical admission procedure. Swabs were analysed at the Institute of Virology at the University of Würzburg and our research laboratory lab of the Department of Gynaecology using reverse transcriptase quantitative polymerase chain reaction (RT-qPCR) or RT-PCR respectively. To determine a past infection with SARS-CoV-2, enzyme-linked immunosorbent assay (ELISA) of immunoglobulins G, A and M were performed with blood serum samples according to manufacturer’s instructions (EL-2006-9601 G, EL-2606-9601-2 M, EL-2606-9601A, Euroimmun, Lübeck, Germany), in the research lab of the Department of Gynaecology.

### Data management

Information on the patient’s state of health, course of breast cancer, and result of the distress thermometer at initial diagnosis [20] were taken from the hard copy medical records, which had been stored in the in the doctor’s office and document archive of the oncological section at the Department of Obstetrics and Gynaecology, University Hospital Würzburg. All participants completed the questionnaires by hand during their therapy stay. To determine the subtype of breast cancer, the following surrogate definitions were used: Luminal A = hormone receptor (HR) positive, Her2 negative, Ki67 ≤ 25%; Luminal B Her2 negative = HR positive, Her2 negative, Ki67 > 25%; Luminal B Her2 positive = HR positive, Her2 positive; Her2 overexpression = HR negative, Her2 positive; Triple negative = HR negative, Her2 negative Leitlinienprogramm Onkologie (Deutsche Krebsgesellschaft, Deutsche Krebshilfe, AWMF): [24]. In total, the study participants completed the following self-report questionnaires; the self-designed COVID-19 pandemic questionnaire, the Stress and Coping Inventory (SCI) [19], the National Comprehensive Cancer Network® (NCCN®) Distress Thermometer (DT) [20], the European Organization for Research and Treatment of Cancer (EORTC) QLQ C30 and the BR23 [17, 21].

All data from the questionnaires and the medical records mentioned in the previous paragraph were entered into an excel datasheet under the corresponding study number, and analysed with SPSS Statistics 26 (IBM). For one patient, information regarding children was missing. In the questionnaires, some individual questions were not answered, either because the patients overlooked the question or were not willing to answer.

### Statistical analysis

Data were tested for normal distribution by Kolmogorov-Smirnov and Shapiro-Wilk tests. Data are presented as median (interquartile range) or mean (± standard deviation). Friedman rank test, Wilcoxon test, Mann-Whitney-U-test and Kruskal-Wallis tests were performed accordingly. *P*-values lower than 0.05 were considered statistically significant. As this was an exploratory study, no correction for multiple comparisons was applied. To test the score reliability, Cronbach’s alpha was calculated. The Spearman’s rho test was performed to test inter-scale correlation. Missing data were identified and categorised accordingly, therefore no data were excluded. The software SPSS Statistics 26 (IBM) was used to perform statistical analyses, create figures and compile tables.

## Results

### Basic characteristics of the study population

Eighty-two breast cancer patients between the ages of 31 and 76 years participated in the study. The study population consisted of 26 breast cancer patients undergoing adjuvant therapies, 26 breast cancer patients undergoing neoadjuvant therapies, and 30 patients undergoing palliative care. The distribution of current breast cancer stages according to the Union for International Cancer Control (UICC), and the subtype of breast cancer according to clinical surrogate definition, are shown in Table 2. The mean age of the female 82 breast cancer patients was 54.63 (±11.10) years. There were no significant differences in age between the different therapy statuses (*p* = 0.212); the mean age of patients undergoing adjuvant therapies was 58.00 (±11.73) years, neoadjuvant therapies 51.77 (±11.69) years, and palliative therapies 54.20 (±9.51) years. Other sociodemographic data are summarised in Table 3. In the analysis of comorbidities, 4/82 breast cancer patients (4.88%) suffered from a previous mental disorder. One patient was undergoing adjuvant therapy, and three were undergoing palliative care. We analysed medication as a proxy for other concomitant diseases, and there was an equal distribution between breast cancer patients undergoing adjuvant, neoadjuvant and palliative therapies.

**Table 2:** Crosstable of the number of patients regarding the current cancer stage according to the Union for International Cancer Control (UICC) as well as the subtype of breast cancer according to a clinical surrogate definition in numbers. Percentages (%) were calculated for the sum of each row and column

		Subtype of Breast Cancer					All
		Luminal A	Luminal B Her2 neg.	Luminal B Her2 pos.	Her2 overexpression	Triple negative	
Current Cancer Stage (UICC)	0	0	0	1	0	0	1 (1.22%)
	1A	6	9	5	0	2	22 (26.83%)
	2A	4	4	5	1	2	16 (19.51%)
	2B	1	3	2	1	1	8 (9.76%)
	3A	1	0	0	0	0	1 (1.22%)
	3B	0	1	0	2	0	3 (3.66%)
	3C	1	0	0	0	0	1 (1.22%)
	4	7	13	9	0	1	30 (36.59%)
all		20 (24.39%)	30 (36.59%)	22 (26.82%)	4 (4.88%)	6 (7.31%)	82 (100%)

**Table 3:** Crosstable of the study population in numbers and percent of each row (beside) and column (below) regarding social demographic data and the therapy status

		Adjuvant therapy *n* = 26	Neoadjuvant therapy *n* = 26	Palliative therapy *n* = 30	All patients	All patients	Missing values
Civil status	Single	7 (36.8%) (26.9%)	4 (21.1%) (15.4%)	8 (42.1%) (26.7%)	19 (23.2%)	82	0
	Married or long-term relationship	19 (30.1%) (73.1%)	22 (34.9%) (84.6%)	22 (34.9%) (73.3%)	63 (76.8%)		
Children	No	4 (21.1%) (15.4%)	4 (21.1%) (16.0%)	11 (57.8%) (36.7%)	19 (23.5%)	81	1
	Yes	6 (28.6%) (23.1%)	9 (42.8%) (36.0%)	6 (28.6) (20.0%)	21 (25.9%)		
	Yes, already grown up	16 (39.0%) (61.5%)	12 (29.2%) (48.0%)	13 (31.7%) (43.3%)	41 (50.6%)		

### Infection status

According to the results from the first part of the COVID-19 pandemic questionnaire, 11 breast cancer patients (13.4%) reported an infection within the last 4 months. Ten suffered from respiratory tract infections and one from a urinary tract infection. 8/11 reported infections were diagnosed before the German lockdown on 16th March 2020. All throat swabs taken during routine clinical practice were negative for SARS-CoV-2. Levels of SARS-CoV-2 antibodies (immunoglobulin G, M, and A) were determined for all study patients. Only one patient had clinically relevant positive antibodies (IgG, IgA as well as IgM), but could not remember experiencing symptoms typical of a COVID-19 infection.

### COVID-19 pandemic questionnaire

**Fig. 1:**
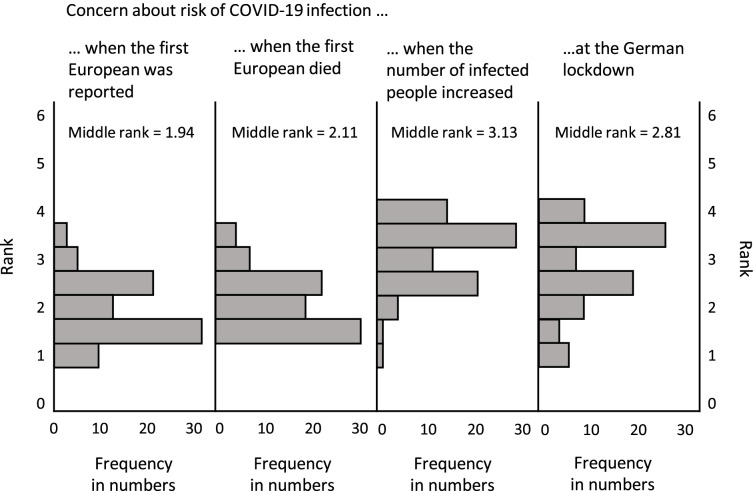
Two-factor analysis of variance for Friedman ranks in related samples of questions 5–8 (*n* = 79) of the COVID-19 pandemic questionnaire. *p*-value = 0.001

Table 1 represents the median values of the second part of the COVID-19 pandemic questionnaire for all breast cancer patients. The maximum median value was 3.00 (2.00–4.00), representing a high concern regarding the impact of the COVID-19 pandemic on life quality. Cronbach’s alpha was 0.88 for the “concern scale”, 0.86 for the “concern over time scale”, and 0.81 for the “impairment scale”. Questions 5–8 related to the timing of the pandemic and its associate restrictions. Figure 1 shows the significant results of the Friedman rank test (*p* = 0.001). Concerns significantly increased during three consecutive time points; time point “the first European patient got ill”, time point “the first European died” (*p* = 0.047), and time point “the number of SARS-CoV-2 infected persons raised” (*p* ≤ 0.0001). There was also a significant decrease in concern at the time point “German lockdown on March 16^th^ 2020” (*p* = 0.019). All values of questions 5–8 significantly differ in the Wilcoxon test. There were no significant differences between adjuvant, neoadjuvant and palliative patients for sums of “concern scale” (10.00 [8.00–13.00] versus (vs.) 10.50 [8.50–14.00] vs. 10.00 [8.00–14.00]; *p* = 0.713), “concern over time scale” (8.00 [7.00–12.00] versus (vs.) 9.00 [8.00–12.00] vs. 9.00 [7.00–12.00]; *p* = 0.760), “impairment scale” (7.50 [5.00–10.00] versus (vs.) 8.00 [6.00–11.00] vs. 8.00 [7.00–10.00]; *p* = 0.977), and the median values of each question regarding COVID-19 worries. In addition, there were no significant differences between age (< 55 years and ≥ 55 years), civil status (single vs. married or long-term relationship), or children (no children vs. children vs. children already grown up) (data not shown).

### Stress and coping inventory (SCI)

We used the stress and coping inventory to analyse stress, concern and coping strategies. For all participants, “stress caused by insecurity” was 19.00 (11.00–26.00), “stress caused by being overwhelmed” was 15.00 (10.00–20.00), and “stress caused by loss” was 11.00 (9.00–16.00). The mean value of “stress symptoms” was 23.00 (20.00–28.00). The value of coping strategies was 11.00 (10.00–12.00) for “positive coping”, 11.00 (9.00–12.00) for “active coping”, 13.00 (12.00–15.00) for “coping by support”, 10.00 (7.00–12.00) for “coping by believing in God or powers that be”, and 5.00 (4.00–7.00) for “coping by drinking alcohol and/or smoking”. Cronbach’s alphas were between 0.598 and 0.847 for 4/5 scales. The scale “coping by drinking alcohol and/or smoking” had a Cronbach’s alpha of 0.456. The inter-scale correlation is summarised in supplemental Table S1.

The SCI revealed that there was a significant difference in “positive coping” between different therapy regimens (*p* = 0.049; Kruskal-Wallis-Test). Breast cancer patients undergoing adjuvant therapy had significantly more positive coping strategies (12.00 [11.00–13.00]) than patients undergoing neoadjuvant therapy (11.00 [9.50–12.00]; *p* = 0.020). There were no other significant differences between the three therapy groups (Table 4).

**Table 4:** Results of the SCI. * Kruskal-Wallis-Test

	Adjuvant therapy *n* = 26		Neoadjuvant therapy *n* = 26		Palliative therapy *n* = 30		
	Median	Interquartile range	Median	Interquartile range	Median	Interquartile range	*p*-value*
Stress caused by unsecurity	14.00	9.00–24.00	19.00	13.00–25.00	22.00	13.00–26.50	0.305
Stress caused by being overwhelmed	13.00	9.00–20.00	13.00	9.00–20.00	17.00	12.00–20.00	0.489
Stress caused by loss	11.00	8.00–15.00	10.00	9.00–17.00	13.50	10.50–16.50	0.382
Stress symptoms	22.50	20.00–27.00	24.00	18.00–28.00	25.50	21.00–30.50	0.439
Positive coping	12.00	11.00–13.00	11.00	9.50–12.00	11.00	9.00–12.00	0.049
Active coping	11.00	9.00–12.00	11.00	8.50–12.00	11.00	10.00–12.50	0.857
Coping by support	13.50	12.00–15.00	13.00	12.00–16.00	13.00	11.50–15.00	0.797
Coping by believing in God or powers that be	11.00	9.00–12.00	8.00	7.00–12.00	11.50	7.50–12.50	0.334
Coping by drinking alcohol and/or smoking	7.00	4.00–7.00	5.00	4.00–7.00	5.00	4.00–6.00	0.481

*p*-Value of Mann-Whitney-U-test of the sum scale „positive coping “equaled *p* = 0.020 between the adjuvant and neoadjuvant patients

According to age, patients younger than 55 years had significantly more “stress caused by insecurity” and “stress caused by being overwhelmed” than the older patients (23.00 [15.00–28.00] vs. 14.50 [9.00–21.50]; *p* = 0.019 and 18.00 [13.00–20.00] vs. 12.00 [9.00–16.50]; *p* = 0.014 respectively). “Stress caused by loss” and stress symptoms were slightly but not significantly higher for patients younger than 55 years (13.00 [10.00–18.00] vs. 10.50 [8.50–14.50], *p* = 0.061 and 26.00 [21.00–28.00] vs. 22.50 [18.00–28.00], *p* = 0.181 respectively). Regarding coping strategies, there were no significant differences between patients younger than 55 years and older patients (data not shown).

Single patients had significantly lower stress symptoms (21.00 [18.00–22.00]) than patients that were married or in a long-term relationship (25.00 [21.00–30.00], *p* = 0.004). However, “coping by support” was significantly higher in married patients or those in a long-term relationship (12.00 [10.00–14.00] vs. 14.00 [12.00–15.00], *p* = 0.039). No other scales of the SCI differed significantly in relation to civil status (data not shown).

Regarding children (no children vs. children vs. children already grown up), there were significant differences in “stress caused by insecurity” and “stress caused by being overwhelmed” between the stress groups (21.00 [12.00–30.00] vs. 25.00 [20.00–30.00] vs. 15.00 [9.00–21.00], *p* = 0.014 and 16.50 [11.00–22.00] vs. 20.00 [18.00–22.00] vs. 12.00 [9.00–16.00], *p* = 0.003 respectively). Patients with children reported significantly more stress than patients with grown up children (“stress caused by insecurity” 25.00 [20.00–30.00] vs. 15.00 [9.00–21.00], *p* = 0.003, “stress caused by being overwhelmed” 20.00 [18.00–22.00] vs. 12.00 [9.00–16.00], *p* = 0.001, “stress caused by loss” 15.00 [10.00–20.00] vs. 10.00 [8.00–16.00], *p* = 0.019). There were no significant differences in stress symptoms between coping strategies (data not shown).

### Distress thermometer (DT)

The median of the NCCN® distress thermometer (DT) at initial breast cancer diagnosis before German lockdown was 5.00 (4.00–7.00) and did not differ significantly from the median of DT after German lockdown (5.00 [3.00–7.00], *p* = 0.260, Wilcoxon test). There were no significant differences in DT score for any subgroup (age, civil status, children, therapy status) between pre- and post-lockdown.

### EORTC QLQ-C30 and QLQ-BR23

For all patients, median “global health status/quality of life (QL2)” was 58.33 (50.00–75.83)%, “physical functioning (PF2)” was 73.33 (60.00–93.33)%, “role functioning (RF)” was 66.67 (33.33–83.33)%, “emotional functioning (EF)” was 58.33 (41.67–75.00)%, “cognitive functioning (CF)” was 83.33 (50.00–100.00)%, “social functioning (SF)” was 66.67 (33.33–83.33)%, “fatigue (FA)” was 44.44 (22.22–66.67)%, “nausea and vomiting (NV)” was 0.00 (0.00–0.00)%, “pain (PA)” was 16.67 (0.00–50.00)%, “dyspnoea (DY)” was 0.00 (0.00–33.33)%, “insomnia (SL)” was 33.33 (0.00–66-67)%, “appetite loss (AP)” was 0.00 (0.00–33.33)%, “constipation (CO)” was 0.00 (0.00–33.33)%, “diarrhoea (DI)” was 0.00 (0.00–0.00)%, “financial difficulties (FI)” was 0.00 (0.00–33.33), “body image (BRBI)” was 66.67 (50.00–91.67)%, “sexual functioning (BRSEF)” was 16.67 (0.00–33.33)%, sexual enjoyment (BRSEE)” was 66.67 (33.33–100.00 ± 34.71)%, “future perspective (BRFU)” was 33.33 (0.00–66.67)%, “systemic therapy side effects (BRST)” was 28.57 (14.29–47.62)%, “breast symptoms (BRBS)” was 8.33 (0.00–25.00), “arm symptoms (BRAS)” was 22.22 (0.00–33.33) and “upset by hair loss (BRHL) was 66.67 (33.33–66.67). Cronbach’s alpha was between 0.666 and 0.902 with two exceptions (NV = 0.045 and BRST = 0.591). Supplemental Table S2 summarises the Spearman’ s rho test, showing the correlation between each item of EORTC QLQ-C30 and QLQ-BR23.

There were no significant differences in most of the items of EORTC QLQ-C30 and QLQ-BR23 with regards to children (no children vs. children vs. children, already grown up), civil status (single vs. married or long-term relationship) or age. However, physical functioning was significantly better (91.67 [66.67–100.00]%) for single patients than for those who were married or in a long-term relationship (73.33 [60.00–86.67]%, *p* = 0.023). Financial difficulties were significantly increased for patients younger than 55 years compared to the older patients (33.33 [0.00–66.67]% vs. 0.00 [0.00–33.33]%, *p* = 0.019).

Table 5 represents the comparison of patients undergoing different therapy types. There were significant differences in RF, SF, FA, PA and AL between patients undergoing adjuvant, neoadjuvant and palliative therapies. RF was highest for patients undergoing adjuvant therapy, in comparison to neoadjuvant and palliative therapies (83.33 [66.67–100.00]% vs. 66.67 [33.33–100.00]% vs. 66.67 [33.33–66.67]%, *p* = 0.035). The same pattern was observed for SF (66.67 [50.00–100.00]% vs. 66.67 [50.00–66.67]% vs. 33.33 [16.67–66.67]%, *p* = 0.044). Palliative patients had the most complaints regarding FA (27.78 [11.11–55.56]% vs. 33.33 [33.33–55.56] vs. 55.56 [33.33–77.78], *p* = 0.023), PA (16.67 [0.00–33.33] vs. 8.33 [0.00–33.33]% vs. 33.33 [16.67–66.67]%, *p* = 0.006) and AL (0.00 [0.00–0.00] vs. 0.00 [0.00–0.00] vs. 0.00 [0.00–66.67], *p* = 0.021). Mann-Whitney testing further revealed significant differences between adjuvant and palliative patients (RF *p* = 0.011; SF *p* = 0.012, FA *p* = 0.009, PA *p* = 0.015, AL *p* = 0.014), and neoadjuvant and palliative patients (PA *p* = 0.004; AL p = respectively 0.042).

**Table 5:** Results of the EORTC QLQ-C30 and QLQ_BR in percent of all breast cancer patients undergoing adjuvant, neoadjuvant or palliative therapies. * Kruskal-Wallis-Test

	Adjuvant therapy *n* = 26		Neoadjuvant therapy *n* = 26		Palliative therapy *n* = 30		
	Median	Interquartile range	Median	Interquartile range	Median	Interquartile range	*p*-value*
Global health status/Quality of Life	58.33	50.00–75.00	62.50	41.67–75.00	50.00	50.00–66.67	0.396
Physical functioning	86.67	66.67–93.33	80.00	66.67–93.33	66.67	46.67–86.67	0.520
Role functioning	83.33	66.67–100.00	66.67	33.33–100.00	66.67	33.33–66.67	0.035
Emotional functioning	58.33	50.00–83.33	58.33	50.00–75.00	50.00	33.33–66.67	0.200
Cognitive functioning	83.33	66.67–100.00	83.33	50.00–100.00	66.67	50.00–83.33	0.318
Social functioning	66.67	50.00–100.00	66.67	50.00–66.67	33.33	16.67–66.67	0.044
Fatigue	27.78	11.11–55.56	33.33	33.33–55.56	55.56	33.33–77.78	0.023
Nausea and vomiting	0.00	0.00–0.00	0.00	0.00–16.67	0.00	0.00–16.67	0.179
Pain	16.67	0.00–33.33	8.33	0.00–33.33	33.33	16.67–66.67	0.006
Dyspnoea	0.00	0.00–33.33	0.00	0.00–33.33	33.33	0.00–66-67	0.840
Insomnia	66.67	0.00–66.67	33.33	0.00–66.67	33.33	33.33–66.67	0.745
Appetite loss	0.00	0.00–0.00	0.00	0.00–0.00	0.00	0.00–66.67	0.021
Constipation	0.00	0.00–33.33	0.00	0.00–0.00	0.00	0.00–33.33	0.187
Diarrhoea	0.00	0.00–0.00	0.00	0.00–33.33	0.00	0.00–0.00	0.355
Financial difficulties	0.00	0.00–33.33	0.00	0.00–33.33	33.33	0.00–66.67	0.264
Body image	66.67	54.17–87.50	75.00	58.33–91.67	62.50	41.67–75.00	0.167
Sexual functioning	8.33	0.00–41.67	16.67	0.00–16.67	16.67	0.00–33.33	0.924
Sexual enjoyment	66.67	50.00–100.00	50.00	16.67–66.67	66.67	33.33–100.00	0.378
Future perspective	33.33	0.00–66.67	33.33	0.00–66.67	33.33	0.00–66.67	0.824
Systemic therapy side effects	21.43	12.70–35.71	38.10	14.29–47.62	30.95	19.05–47.62	0.295
Breast symptoms	8.33	0.00–25.00	0.00	0.00–16.67	4.17	0.00–25.00	0.398
Arm symptoms	22.22	11.11–44.44	22.22	0.00–33.33	11.11	0.00–33.33	0.488
Upset by hair loss	33.33	0.00–50.00	66.67	0.00–100.00	66.67	66.67–66.67	0.470

### Groups by concern

We did not observe any significant differences in pandemic-related stress levels between patients undergoing different therapy regimes. We therefore stratified patients based on their concerns regarding the COVID-19 pandemic. The minimum value of an answer on the COVID-19 pandemic scale was 1, and the maximum was 5. Questions 1–4 mainly represented concerns about risk of infection with COVID-19. The range of the sum of these four answers was 4 to 20 and allowed stratification into three groups; 27 patients (32.9%) with no concerns/only thoughts (sum range 4–8), 30 patients (36.6%) with a little concern (sum range 9–12), and 23 patients (28.0%) with concerns often/all the time.

This stratification was replicated using the SCI, revealing significant differences between these groups regarding stress caused by insecurity, being overwhelmed, loss, and stress symptoms (Table 6). The current DT further supported these significant differences. Patients with no concerns/only thoughts score had a DT value of 3.00 (2.00–5.00), patients with a little concern a value of 5.50 (3.50–7.00), and patients with concerns often/all the time scored 6.00 (5.00–7.00) (*p* ≤ 0.0001). Mann-Whitney U testing for group comparisons also revealed significant differences; no concerns/only thoughts versus a little concern had a *p*-value of 0.009, and no concerns/only thoughts versus concerns often/all the time had a *p*-value of ≤0.0001.

**Table 6:** Results of the SCI stratified by groups of concern. * Kruskal-Wallis-Test

	No/thoughts *n* = 27		A little *n* = 30		Often/all the time *N* = 23		
	Median	Interquartile range	Median	Interquartile range	Median	Interquartile range	*p*-value*
Stress caused by unsecurity	11.00	8.00–18.00	19.00	13.00–25.00	25.50	20.50–30.00	0.000
Stress caused by being overwhelmed	10.50	7.00–13.00	16.00	11.00–19.00	20.50	17.00–23.00	0.000
Stress caused by loss	9.00	7.00–11.00	11.00	10.00–17.00	15.00	12.00–19.00	0.001
Stress symptoms	20.50	16.50–23.00	24.00	20.00–28.00	28.00	24.00–31.00	0.000
Positive coping	11.00	9.00–13.00	11.00	10.00–12.00	12.00	8.00–13.00	0.896
Active coping	11.00	8.00–14.00	11.00	10.00–12.00	11.00	10.00–13.00	0.615
Coping by support	13.50	10.00–16.00	13.00	12.00–15.00	13.00	12.00–15.00	0.713
Coping by believing in God or powers that be	11.00	7.00–13.00	10.00	8.00–12.00	10.00	7.00–12.00	0.863
Coping by drinking alcohol and/or smoking	5.00	4.00–7.00	5.00	4.00–7.00	6.00	4.00–7.00	0.946

Table 7 shows the results of these three stratified subgroups for the EORTC QLQ-C30 and QLQ-BR23 questionnaires. The data revealed that the more COVID-19-related concern that was expressed, the lower the QL2, PF, RF, EF, CF, SF, BRBI and BRFU scores. These correlations were all statistically significant, with the exception of PF. Higher concerns were additionally associated with increased scores for FA, PA, DY, SL, CO, FI, BRST and BRAS. However, only, the associations with increased FA, SL and BRST were significant.

**Table 7:** Results of the EORTC QLQ-C30 in percent of all breast cancer patients grouped by subgroups of concerns. * Kruskal-Wallis test

Concern about COVID-19	No/thoughts *n* = 27		A little *n* = 30		Often/all the time *n* = 23		
	Median	Interquartile range	Median	Interquartile range	Median	Interquartile range	*p*-value*
Global health status/Quality of Life	58.33	50.00–75.00	66.67	50.00–83.33	50.00	41.67–58.33	0.050
Physical functioning	80.00	66.67–93.33	75.00	60.00–93.33	66.67	46.67–86.67	0.196
Role functioning	66.67	50.00–100.00	66.67	66.67–83.33	50.00	33.33	0.007
Emotional functioning	77.78	58.33–100.00	50.00	33.33–66.67	45.83	41.67–58.33	0.000
Cognitive functioning	83.33	66.67–100.00	83.33	50.00–100.00	50.00	50.00–83.33	0.001
Social functioning	83.33	50.00–100.00	50.00	33.33–66.67	50.00	16.67–66.67	0.002
Fatigue	33.33	11.11–55.56	44.44	22.22–55.56	55.56	33.33–77.78	0.032
Nausea and vomiting	0.00	0.00–16.67	0.00	0.00–0.00	0.00	0.00–0.00	0.827
Pain	0.00	0.00–33.33	33.33	0.00–50.00	33.33	0.00–66.67	0.276
Dyspnoea	0.00	0.00–33.33	0.00	0.00–33.33	33.33	0.00–66.67	0.308
Insomnia	16.67	0.00–33.33	66.67	33.33–100.00	66.67	33.33–66.67	0.001
Appetite loss	0.00	0.00–33.33	0.00	0.00–33.33	0.00	0.00–33.33	0.938
Constipation	0.00	0.00–0.00	0.00	0.00–33.33	0.00	0.00–66.67	0.118
Diarrhoea	0.00	0.00–33.33	0.00	0.00–0.00	0.00	0.00–0.00	0.594
Financial difficulties	0.00	0.00–33.33	16.67	0.00–50.00	33.33	0.00–33.33	0.155
Body image	75.00	58.33–83.33	66.67	50.00–91.67	54.17	16.67–75.00	0.013
Sexual functioning	8.33	0.00–33.33	16.67	0.00–33.33	0.00	0.00–33.33	0.216
Sexual enjoyment	33.33	0.00–66.67	66.67	33.33–100.00	66.67	33.33–100.00	0.273
Future perspective	66.67	33.33–66.67	33.33	0.00–66.67	0.00	0.00–33.33	0.000
Systemic therapy side effects	20.63	9.52–38.10	33.33	19.05–47.62	33.33	19.05–47.62	0.020
Breast symptoms	0.00	0.00–16.67	8.33	0.00–50.00	8.33	0.00–33.33	0.348
Arm symptoms	11.11	0.00–22.22	22.22	0.00–33.33	22.22	0.00–44.44	0.296
Upset by hair loss	33.33	33.33–66.67	33.33	0.00–66.67	66.67	66.67–100.00	0.116

## Discussion

The COVID-19 pandemic is a public health emergency of international concern that presents economies and health care systems with new challenges. Economical, material and physical problems, along with the danger to public health and severe restrictions on public life, is putting great pressure on large proportions of the population regarding mental health and psychological resilience. For patients with cancer, the diagnosis, treatment and tumour follow-up is already commonly associated with increased levels of psychological distress [25]. The combination of both the COVID-19 pandemic and cancer diagnosis therefore constitutes an enormous psychological burden [26]. Previous literature has demonstrated that women currently or previously treated for breast cancer have an increased risk of depression and psychological problems compared to other types of cancer [27, 28]. For breast cancer patients, social and psychological support is therefore particularly crucial for supporting quality of life (QoL) and mental health [29].

Research data to identify and define groups of oncological patients, that need evidence-based strategies to reduce adverse psychological impacts and psychiatric symptoms during the COVID-19 pandemic is currently limited, particularly breast cancer patients [13, 15]. Defining subgroups of breast cancer patients with increased need for psychological support could improve adherence to treatment, enhance cancer survival, and reduce treatment costs [30].

### The COVID-19 pandemic and stress

The aim of this study was to improve the understanding of psychological stress levels in breast cancer patients during the initial stage of the COVID-19 pandemic, and to identify critical subgroups who may need appropriate and personalised psychological interventions. The screening tool used to determine psychological stress in breast cancer patients was the distress thermometer. As distress levels are routinely clinically assessed in all breast cancer patients during treatment we were able to compare data from before the pandemic to data collected during the pandemic (April to June 2020) in our study group.

Surprisingly, we did not detect an increase in distress levels resulting from COVID-19 and the associated lockdown measures. It is possible that the unique types of distress caused by the COVID-19 pandemic are not adequately reflected in the distress thermometer, and therefore caution is advised when interpreting these results. However, the results suggest that despite the COVID-19 pandemic, distress levels did not deteriorate since the first evaluation of distress at time of diagnosis. One possible explanation for this finding might be that distress levels and life quality tend to improve during the course of disease [29, 31, 32]. In the present study, there were several participants whose first distress evaluation at the time of diagnosis was up to 2 years before the second evaluation during the initial phase of COVID-19 (April to June 2020).

Furthermore, it is possible that lockdown measures, home-office, and the reduction of social duties have also contributed to decreased distress levels, as some tumour patients perceive normal participation in social life as an increased stress [29]. Many breast cancer patients are additionally strongly impacted by physical problems, body image disruptions and side effects of cancer therapy, therefore a perceived decrease in social obligations may have further eased stress [29, 33].

Lastly, the observed stable distress levels during COVID-19 pandemic could be due to the continuation of cancer therapy. Sufficient medical resources and the limited number of COVID-19 patients in our facility (University Hospital of Würzburg), meant there were no treatment delays or interruptions for our cancer patients.

### Quality of life in the COVID-19 pandemic

In addition to distress comparisons pre- and during the pandemic, we also correlated quality of life for our participants (assessed by the self-report questionnaires EORTC QLQ-C30 and QLQ-BR23) with reference data published by the EORTC [34]. We detected a deterioration in Global health status, Physical functioning, Role functioning, Emotional functioning, Cognitive functioning and Social functioning [34]. In contrast to the distress thermometer where distress is summarised in one numeric value, the differentiated analysis of the EORTC QLQ-C30 showed a significant effect of the COVID-19 pandemic on distress levels in comparison to the reference data. This finding is in contrast to the results published by Bargon et al. They found that during the COVID-19 pandemic, there was a small significant increase in Quality of Life, Physical Functioning, Role functioning and Social functioning, but a significant decrease in Emotional functioning during the COVID-19 pandemic, compared to 2 years prepandemic. However, in this study the population consisted of breast cancer patients and survivors, which could potentially explain the different results [29]. As we did not measure life quality with the EORTC QLQ-C30 in our participants prepandemic, we cannot present a direct before/after comparison for this parameter. However, a previous meta–analysis clearly demonstrated that patients affected by breast cancer are particularly vulnerable to feelings of loneliness, anxiety, and physical integrity when compared to unaffected controls [27]. Due to social isolation measures and the extent of concern regarding the pandemic, it is highly likely that life quality will have been negatively affected for some tumour patients.

### Stress and subgroups of BC patients

In addition to the already described questionnaires, we also used the Stress and Coping Inventory (SCI) to measure stress symptoms, stress burden and coping strategies [18, 19]. The aim of using several different questionnaires determining stress levels was to identify subgroups of breast cancer patients with specific clinical parameters who may be particularly vulnerable to mental stress, and therefore need additional psychological care.

We examined possible correlations between stress levels and different clinical parameters such as age, social status and therapy regimen. However, we could not identify a subgroup of breast cancer patients who were significantly more susceptible to stress than others. The measured stress levels were distributed almost equally between the clinical subsets (> 55 years and below; single or in a relationship; children or no children; neoadjuvant, adjuvant or palliative treatment). This was surprising, as we expected certain subgroups (such as patients with little social support or of a higher age) to be more affected by the COVID-19 pandemic and associated lockdown measures than women who are socially well-integrated. Our results are however consistent with the data published by Stark et al., who demonstrated that although anxiety disorders and depression are associated with age and socioeconomic status in the general population, this is not the case for breast cancer patients [35].

### Levels of concerns regarding the COVID-19 pandemic

As we could not identify clinical subgroups of breast cancer patients that were significantly more distressed due to the pandemic, we compared groups with different levels of concerns regarding the COVID-19 pandemic. Based on the first four questions of our “COVID-19 pandemic questionnaire” we set up three different subgroups; “no concern / only thoughts”, “little concern” and “concern often / all the time”. Our analysis revealed that many parameters of the EORTC QLQ-C30, EORTC QLQ-BR23, and the SCI were significantly different between the three subgroups of COVID-19 concern. Patients in the subgroup demonstrating “concern often / all the time” had significantly higher scores for psychological stress. This therefore suggests that the four questions included in our self-designed questionnaire are suitable for reliable detection of breast cancer patients who may be particularly susceptible to the increased stress caused by the COVID-19 pandemic and the lockdown measures. Additionally, in contrast to questionnaires with up to 52 parameters, our results suggest that answering just four questions may be highly feasible in clinical practice.

### Limitations of the study

There are several limitations of this study. One of this limitations of this study was the small sample size. Additionally, self-reported results might not always accurately reflect the levels of psychological impact in cancer patients. We only had pre-pandemic data for one questionnaire, and therefore could only directly compare pre- and during pandemic results for one dataset. The COVID-19 stress questionnaire was self-designed, which could also be deemed a limitation. No questionnaires were used to detect clinical depression or anxiety, and only clinical diagnoses were recorded. Lastly, only basic sociodemographic data was assessed. Further information regarding socioeconomic status, race, ethnicity, cultural background, and religion were not collected. We therefore cannot assess the influence of these possible confounding factors.

## Conclusion

To our knowledge, this is the first analysis that compared the levels of distress within a defined patient group (breast cancer patients) before the spread of SARS-CoV-2 and after the German lockdown measures. Additionally, this study compares different self-questionnaires in terms of their ability to detect mental stress in a group of patients undergoing active breast cancer therapy. Due to our “COVID-19 pandemic questionnaire”, we were able to identify a subgroup of psychologically susceptible breast cancer patients with only modest effort. This subgroup reported “concern often / all the time” in our questionnaire and showed significantly higher levels of distress and lower quality of life. The identification of this subgroup may facilitate personalised interventions and treatments, such as web-based psychosocial interventions and therapies, which have been shown as promising during a pandemic situation [36, 37].

## Supplementary Information

**Additional file 1: Supplemental Figure 1.** Flow chart of the procedure for recruitment of participants and data collection.

**Additional file 2: Supplemental Table S1.** Correlation of the different scales of the stress and coping inventory. p = two-sided significance; * *p* < 0.05; ** *p* < 0.01.

**Additional file 3: Supplemental Table S2.** Correlation of the different scales of the EORTC QLQ C30 and BR23. p = two-sided significance; * *p* < 0.05; ** *p* < 0.01. QL2 = Global health status/Quality of Life.

## Data Availability

The datasets generated and/or analysed during the current study are not publicly available due to privacy policy but are available from the corresponding author on reasonable request within the European Union or countries with an appropriate statutory level of data protection and cooperation agreements.

## Abbreviations

COVID-19: Infection by Coronavirus SARS-CoV-2
SCI: Stress and Coping Inventory
DT: Distress Thermometer of the National Comprehensive Cancer Network® (NCCN®)
EORTC: European Organisation for Research and Treatment of Cancer
HR: Hormone Receptor
PCR: Polymerase Chain Reaction
ELISA: Enzyme-Linked Immunosorbent Assay
QoL: Quality of Life
