# Supplementary material for: The effects of the COVID-19 pandemic on psychological stress in breast cancer patients
Source: BMC Cancer. 2021 Dec 31;21:1356. doi: 10.1186/s12885-021-09012-y (PMC8719114; doi:10.1186/s12885-021-09012-y)
Supplement: Supplementary file 1 — Additional file 1: Supplemental Figure 1. Flow chart of the procedure for recruitment of participants and data collection. [file 12885_2021_9012_MOESM1_ESM.ppt]

## Slide 1
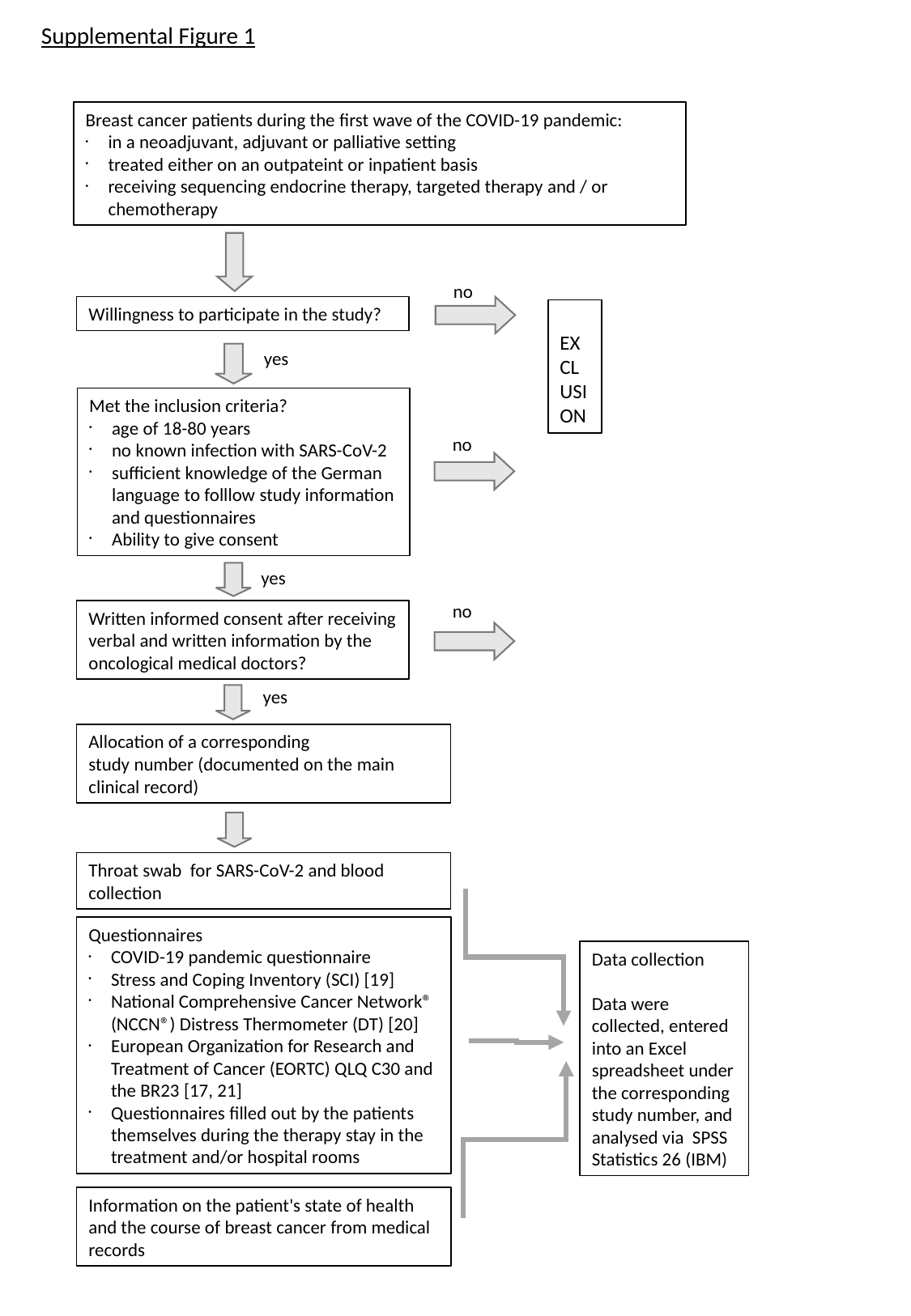

Supplemental Figure 1
Breast cancer patients during the first wave of the COVID-19 pandemic:
in a neoadjuvant, adjuvant or palliative setting
treated either on an outpateint or inpatient basis
receiving sequencing endocrine therapy, targeted therapy and / or chemotherapy
no
Willingness to participate in the study?
 EXCLUSION
yes
Met the inclusion criteria?
age of 18-80 years
no known infection with SARS-CoV-2
sufficient knowledge of the German language to folllow study information and questionnaires
Ability to give consent
no
yes
no
Written informed consent after receiving verbal and written information by the oncological medical doctors?
yes
Allocation of a corresponding
study number (documented on the main clinical record)
Throat swab for SARS-CoV-2 and blood collection
Questionnaires
COVID-19 pandemic questionnaire
Stress and Coping Inventory (SCI) [19]
National Comprehensive Cancer Network® (NCCN®) Distress Thermometer (DT) [20]
European Organization for Research and Treatment of Cancer (EORTC) QLQ C30 and the BR23 [17, 21]
Questionnaires filled out by the patients themselves during the therapy stay in the treatment and/or hospital rooms
Data collection
Data were collected, entered into an Excel spreadsheet under the corresponding study number, and analysed via SPSS Statistics 26 (IBM)
Information on the patient's state of health and the course of breast cancer from medical records
