# Supplementary material for: The effects of the COVID-19 pandemic on psychological stress in breast cancer patients
Source: BMC Cancer. 2021 Dec 31;21:1356. doi: 10.1186/s12885-021-09012-y (PMC8719114; doi:10.1186/s12885-021-09012-y)
Supplement: Supplementary file 2 — Additional file 2: Supplemental Table S1. Correlation of the different scales of the stress and coping inventory. p = two-sided significance; * p < 0.05; ** p < 0.01. [file 12885_2021_9012_MOESM2_ESM.pptx]

## Slide 1
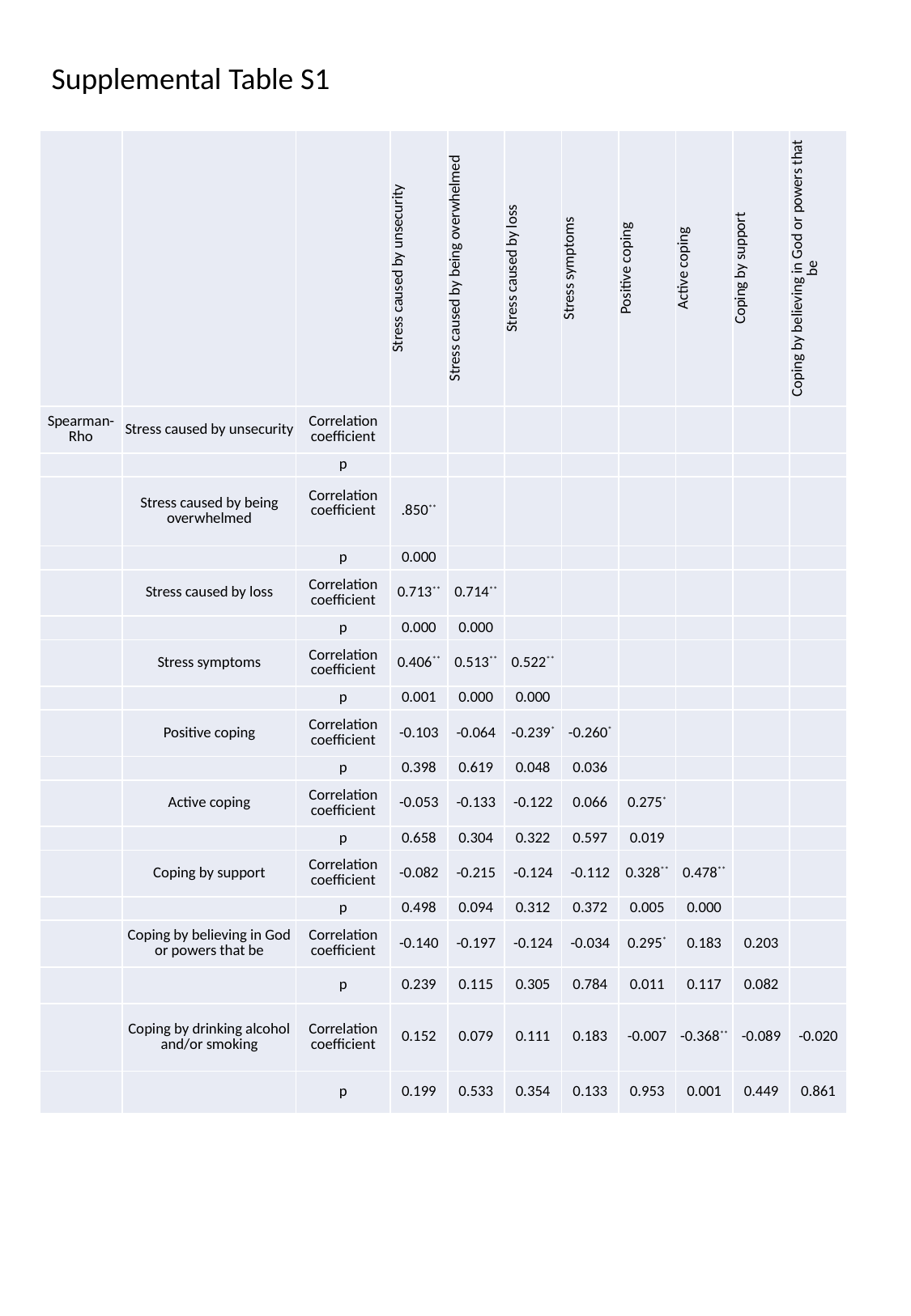

Supplemental Table S1
| | | | Stress caused by unsecurity | Stress caused by being overwhelmed | Stress caused by loss | Stress symptoms | Positive coping | Active coping | Coping by support | Coping by believing in God or powers that be |
| --- | --- | --- | --- | --- | --- | --- | --- | --- | --- | --- |
| Spearman-Rho | Stress caused by unsecurity | Correlation coefficient | | | | | | | | |
| | | p | | | | | | | | |
| | Stress caused by being overwhelmed | Correlation coefficient | .850\*\* | | | | | | | |
| | | p | 0.000 | | | | | | | |
| | Stress caused by loss | Correlation coefficient | 0.713\*\* | 0.714\*\* | | | | | | |
| | | p | 0.000 | 0.000 | | | | | | |
| | Stress symptoms | Correlation coefficient | 0.406\*\* | 0.513\*\* | 0.522\*\* | | | | | |
| | | p | 0.001 | 0.000 | 0.000 | | | | | |
| | Positive coping | Correlation coefficient | -0.103 | -0.064 | -0.239\* | -0.260\* | | | | |
| | | p | 0.398 | 0.619 | 0.048 | 0.036 | | | | |
| | Active coping | Correlation coefficient | -0.053 | -0.133 | -0.122 | 0.066 | 0.275\* | | | |
| | | p | 0.658 | 0.304 | 0.322 | 0.597 | 0.019 | | | |
| | Coping by support | Correlation coefficient | -0.082 | -0.215 | -0.124 | -0.112 | 0.328\*\* | 0.478\*\* | | |
| | | p | 0.498 | 0.094 | 0.312 | 0.372 | 0.005 | 0.000 | | |
| | Coping by believing in God or powers that be | Correlation coefficient | -0.140 | -0.197 | -0.124 | -0.034 | 0.295\* | 0.183 | 0.203 | |
| | | p | 0.239 | 0.115 | 0.305 | 0.784 | 0.011 | 0.117 | 0.082 | |
| | Coping by drinking alcohol and/or smoking | Correlation coefficient | 0.152 | 0.079 | 0.111 | 0.183 | -0.007 | -0.368\*\* | -0.089 | -0.020 |
| | | p | 0.199 | 0.533 | 0.354 | 0.133 | 0.953 | 0.001 | 0.449 | 0.861 |
