# Supplementary material for: The effects of the COVID-19 pandemic on psychological stress in breast cancer patients
Source: BMC Cancer. 2021 Dec 31;21:1356. doi: 10.1186/s12885-021-09012-y (PMC8719114; doi:10.1186/s12885-021-09012-y)
Supplement: Supplementary file 3 — Additional file 3: Supplemental Table S2. Correlation of the different scales of the EORTC QLQ C30 and BR23. p = two-sided significance; * p < 0.05; ** p < 0.01. QL2 = Global health status/Quality of Life. [file 12885_2021_9012_MOESM3_ESM.pptx]

## Slide 1
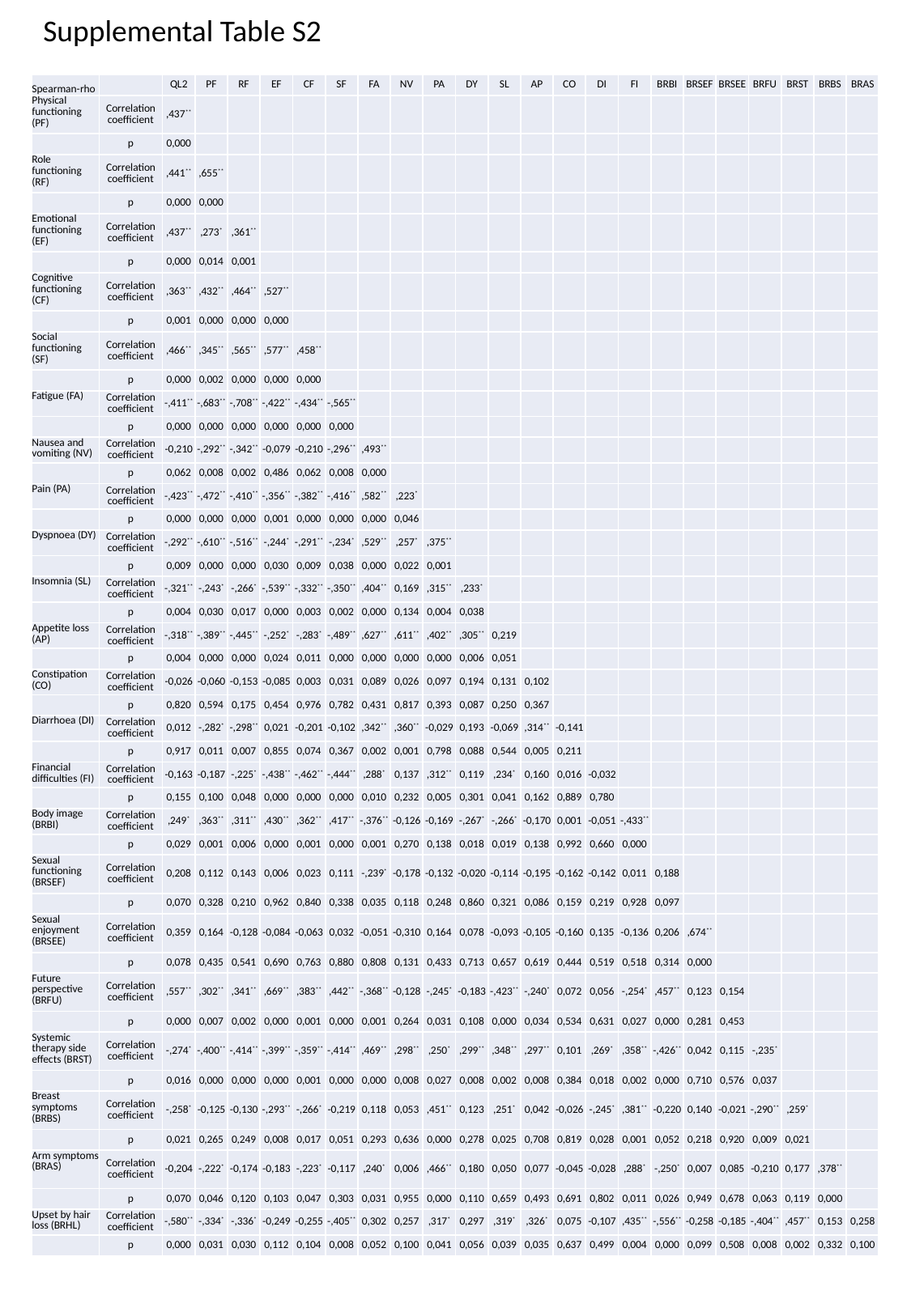

Supplemental Table S2
| Spearman-rho | | QL2 | PF | RF | EF | CF | SF | FA | NV | PA | DY | SL | AP | CO | DI | FI | BRBI | BRSEF | BRSEE | BRFU | BRST | BRBS | BRAS |
| --- | --- | --- | --- | --- | --- | --- | --- | --- | --- | --- | --- | --- | --- | --- | --- | --- | --- | --- | --- | --- | --- | --- | --- |
| Physical functioning (PF) | Correlation coefficient | ,437\*\* | | | | | | | | | | | | | | | | | | | | | |
| | p | 0,000 | | | | | | | | | | | | | | | | | | | | | |
| Role functioning (RF) | Correlation coefficient | ,441\*\* | ,655\*\* | | | | | | | | | | | | | | | | | | | | |
| | p | 0,000 | 0,000 | | | | | | | | | | | | | | | | | | | | |
| Emotional functioning (EF) | Correlation coefficient | ,437\*\* | ,273\* | ,361\*\* | | | | | | | | | | | | | | | | | | | |
| | p | 0,000 | 0,014 | 0,001 | | | | | | | | | | | | | | | | | | | |
| Cognitive functioning (CF) | Correlation coefficient | ,363\*\* | ,432\*\* | ,464\*\* | ,527\*\* | | | | | | | | | | | | | | | | | | |
| | p | 0,001 | 0,000 | 0,000 | 0,000 | | | | | | | | | | | | | | | | | | |
| Social functioning (SF) | Correlation coefficient | ,466\*\* | ,345\*\* | ,565\*\* | ,577\*\* | ,458\*\* | | | | | | | | | | | | | | | | | |
| | p | 0,000 | 0,002 | 0,000 | 0,000 | 0,000 | | | | | | | | | | | | | | | | | |
| Fatigue (FA) | Correlation coefficient | -,411\*\* | -,683\*\* | -,708\*\* | -,422\*\* | -,434\*\* | -,565\*\* | | | | | | | | | | | | | | | | |
| | p | 0,000 | 0,000 | 0,000 | 0,000 | 0,000 | 0,000 | | | | | | | | | | | | | | | | |
| Nausea and vomiting (NV) | Correlation coefficient | -0,210 | -,292\*\* | -,342\*\* | -0,079 | -0,210 | -,296\*\* | ,493\*\* | | | | | | | | | | | | | | | |
| | p | 0,062 | 0,008 | 0,002 | 0,486 | 0,062 | 0,008 | 0,000 | | | | | | | | | | | | | | | |
| Pain (PA) | Correlation coefficient | -,423\*\* | -,472\*\* | -,410\*\* | -,356\*\* | -,382\*\* | -,416\*\* | ,582\*\* | ,223\* | | | | | | | | | | | | | | |
| | p | 0,000 | 0,000 | 0,000 | 0,001 | 0,000 | 0,000 | 0,000 | 0,046 | | | | | | | | | | | | | | |
| Dyspnoea (DY) | Correlation coefficient | -,292\*\* | -,610\*\* | -,516\*\* | -,244\* | -,291\*\* | -,234\* | ,529\*\* | ,257\* | ,375\*\* | | | | | | | | | | | | | |
| | p | 0,009 | 0,000 | 0,000 | 0,030 | 0,009 | 0,038 | 0,000 | 0,022 | 0,001 | | | | | | | | | | | | | |
| Insomnia (SL) | Correlation coefficient | -,321\*\* | -,243\* | -,266\* | -,539\*\* | -,332\*\* | -,350\*\* | ,404\*\* | 0,169 | ,315\*\* | ,233\* | | | | | | | | | | | | |
| | p | 0,004 | 0,030 | 0,017 | 0,000 | 0,003 | 0,002 | 0,000 | 0,134 | 0,004 | 0,038 | | | | | | | | | | | | |
| Appetite loss (AP) | Correlation coefficient | -,318\*\* | -,389\*\* | -,445\*\* | -,252\* | -,283\* | -,489\*\* | ,627\*\* | ,611\*\* | ,402\*\* | ,305\*\* | 0,219 | | | | | | | | | | | |
| | p | 0,004 | 0,000 | 0,000 | 0,024 | 0,011 | 0,000 | 0,000 | 0,000 | 0,000 | 0,006 | 0,051 | | | | | | | | | | | |
| Constipation (CO) | Correlation coefficient | -0,026 | -0,060 | -0,153 | -0,085 | 0,003 | 0,031 | 0,089 | 0,026 | 0,097 | 0,194 | 0,131 | 0,102 | | | | | | | | | | |
| | p | 0,820 | 0,594 | 0,175 | 0,454 | 0,976 | 0,782 | 0,431 | 0,817 | 0,393 | 0,087 | 0,250 | 0,367 | | | | | | | | | | |
| Diarrhoea (DI) | Correlation coefficient | 0,012 | -,282\* | -,298\*\* | 0,021 | -0,201 | -0,102 | ,342\*\* | ,360\*\* | -0,029 | 0,193 | -0,069 | ,314\*\* | -0,141 | | | | | | | | | |
| | p | 0,917 | 0,011 | 0,007 | 0,855 | 0,074 | 0,367 | 0,002 | 0,001 | 0,798 | 0,088 | 0,544 | 0,005 | 0,211 | | | | | | | | | |
| Financial difficulties (FI) | Correlation coefficient | -0,163 | -0,187 | -,225\* | -,438\*\* | -,462\*\* | -,444\*\* | ,288\* | 0,137 | ,312\*\* | 0,119 | ,234\* | 0,160 | 0,016 | -0,032 | | | | | | | | |
| | p | 0,155 | 0,100 | 0,048 | 0,000 | 0,000 | 0,000 | 0,010 | 0,232 | 0,005 | 0,301 | 0,041 | 0,162 | 0,889 | 0,780 | | | | | | | | |
| Body image (BRBI) | Correlation coefficient | ,249\* | ,363\*\* | ,311\*\* | ,430\*\* | ,362\*\* | ,417\*\* | -,376\*\* | -0,126 | -0,169 | -,267\* | -,266\* | -0,170 | 0,001 | -0,051 | -,433\*\* | | | | | | | |
| | p | 0,029 | 0,001 | 0,006 | 0,000 | 0,001 | 0,000 | 0,001 | 0,270 | 0,138 | 0,018 | 0,019 | 0,138 | 0,992 | 0,660 | 0,000 | | | | | | | |
| Sexual functioning (BRSEF) | Correlation coefficient | 0,208 | 0,112 | 0,143 | 0,006 | 0,023 | 0,111 | -,239\* | -0,178 | -0,132 | -0,020 | -0,114 | -0,195 | -0,162 | -0,142 | 0,011 | 0,188 | | | | | | |
| | p | 0,070 | 0,328 | 0,210 | 0,962 | 0,840 | 0,338 | 0,035 | 0,118 | 0,248 | 0,860 | 0,321 | 0,086 | 0,159 | 0,219 | 0,928 | 0,097 | | | | | | |
| Sexual enjoyment (BRSEE) | Correlation coefficient | 0,359 | 0,164 | -0,128 | -0,084 | -0,063 | 0,032 | -0,051 | -0,310 | 0,164 | 0,078 | -0,093 | -0,105 | -0,160 | 0,135 | -0,136 | 0,206 | ,674\*\* | | | | | |
| | p | 0,078 | 0,435 | 0,541 | 0,690 | 0,763 | 0,880 | 0,808 | 0,131 | 0,433 | 0,713 | 0,657 | 0,619 | 0,444 | 0,519 | 0,518 | 0,314 | 0,000 | | | | | |
| Future perspective (BRFU) | Correlation coefficient | ,557\*\* | ,302\*\* | ,341\*\* | ,669\*\* | ,383\*\* | ,442\*\* | -,368\*\* | -0,128 | -,245\* | -0,183 | -,423\*\* | -,240\* | 0,072 | 0,056 | -,254\* | ,457\*\* | 0,123 | 0,154 | | | | |
| | p | 0,000 | 0,007 | 0,002 | 0,000 | 0,001 | 0,000 | 0,001 | 0,264 | 0,031 | 0,108 | 0,000 | 0,034 | 0,534 | 0,631 | 0,027 | 0,000 | 0,281 | 0,453 | | | | |
| Systemic therapy side effects (BRST) | Correlation coefficient | -,274\* | -,400\*\* | -,414\*\* | -,399\*\* | -,359\*\* | -,414\*\* | ,469\*\* | ,298\*\* | ,250\* | ,299\*\* | ,348\*\* | ,297\*\* | 0,101 | ,269\* | ,358\*\* | -,426\*\* | 0,042 | 0,115 | -,235\* | | | |
| | p | 0,016 | 0,000 | 0,000 | 0,000 | 0,001 | 0,000 | 0,000 | 0,008 | 0,027 | 0,008 | 0,002 | 0,008 | 0,384 | 0,018 | 0,002 | 0,000 | 0,710 | 0,576 | 0,037 | | | |
| Breast symptoms (BRBS) | Correlation coefficient | -,258\* | -0,125 | -0,130 | -,293\*\* | -,266\* | -0,219 | 0,118 | 0,053 | ,451\*\* | 0,123 | ,251\* | 0,042 | -0,026 | -,245\* | ,381\*\* | -0,220 | 0,140 | -0,021 | -,290\*\* | ,259\* | | |
| | p | 0,021 | 0,265 | 0,249 | 0,008 | 0,017 | 0,051 | 0,293 | 0,636 | 0,000 | 0,278 | 0,025 | 0,708 | 0,819 | 0,028 | 0,001 | 0,052 | 0,218 | 0,920 | 0,009 | 0,021 | | |
| Arm symptoms (BRAS) | Correlation coefficient | -0,204 | -,222\* | -0,174 | -0,183 | -,223\* | -0,117 | ,240\* | 0,006 | ,466\*\* | 0,180 | 0,050 | 0,077 | -0,045 | -0,028 | ,288\* | -,250\* | 0,007 | 0,085 | -0,210 | 0,177 | ,378\*\* | |
| | p | 0,070 | 0,046 | 0,120 | 0,103 | 0,047 | 0,303 | 0,031 | 0,955 | 0,000 | 0,110 | 0,659 | 0,493 | 0,691 | 0,802 | 0,011 | 0,026 | 0,949 | 0,678 | 0,063 | 0,119 | 0,000 | |
| Upset by hair loss (BRHL) | Correlation coefficient | -,580\*\* | -,334\* | -,336\* | -0,249 | -0,255 | -,405\*\* | 0,302 | 0,257 | ,317\* | 0,297 | ,319\* | ,326\* | 0,075 | -0,107 | ,435\*\* | -,556\*\* | -0,258 | -0,185 | -,404\*\* | ,457\*\* | 0,153 | 0,258 |
| | p | 0,000 | 0,031 | 0,030 | 0,112 | 0,104 | 0,008 | 0,052 | 0,100 | 0,041 | 0,056 | 0,039 | 0,035 | 0,637 | 0,499 | 0,004 | 0,000 | 0,099 | 0,508 | 0,008 | 0,002 | 0,332 | 0,100 |
